# Effect of Intrapulmonary Gas Composition on Lung Ultrasound A-Line Artifacts and Grayscale Intensity: A Prospective Exploratory Study with Heliox in Healthy Volunteers

**DOI:** 10.3390/jcm15218723

**Published:** 2026-11-08

**Authors:** Małgorzata Leciejewska, Natalia Buda, Mateusz Pawłowski

**Affiliations:** 1 Department of Pulmonology, Wojewódzkie Centrum Szpitalne Kotliny Jeleniogórskiej, Ogińskiego 6, 58-506 Jelenia Góra, Poland; 2 Clinical Ultrasound Laboratory, Medical Simulation Center, Medical University of Gdańsk, Dębinki 7, 80-211 Gdańsk, Poland; natabud@gumed.edu.pl; 3 Faculty of Medicine, Medical University of Gdańsk, Dębinki 7, 80-211 Gdańsk, Poland; pawlowskimateusz10@gmail.com

**Keywords:** lung ultrasound, A-lines, heliox, helium–oxygen mixture, acoustic impedance, ultrasound artifacts, grayscale analysis, emphysema

## Abstract

**Background/Objectives**: Lung ultrasound (LUS) of the aerated lung relies on artifacts generated at the pleural interface, but the contribution of intrapulmonary gas to these artifacts has not been studied in humans. Heliox, a helium–oxygen mixture with low density and acoustic impedance, allows the gas phase of the lung to be modified without altering its structure. We examined whether heliox breathing alters A-line artifacts and grayscale intensity in LUS images. **Methods**: In this prospective exploratory study, 52 healthy volunteers underwent LUS at four thoracic locations during ambient-air breathing and subsequently during heliox (80% He/20% O_2_) breathing. A-lines were counted manually, and grayscale intensity and horizontal reverberations were quantified in ImageJ. Paired differences were analyzed with a clustered Wilcoxon signed-rank test and cluster-bootstrap 95% confidence intervals (CIs). Reliability of A-line counting was assessed in a stratified random subsample (13 participants, 104 images) by blinded test–retest and inter-observer re-counting. **Results**: Heliox breathing increased global grayscale intensity (mean difference 6.7 grayscale units, 95% CI 4.1–9.2; *p* < 0.001), most markedly in the deepest image compartment (10.1 units, 95% CI 6.5–13.9), and the number of visible A-lines (0.45, 95% CI 0.30–0.62; *p* < 0.001). Horizontal reverberations did not differ. In the blinded subsample, the heliox–air difference in A-lines decreased from 0.44 in the original unblinded count to 0.21 in the blinded test–retest and 0.04 in the second reader’s count, and the 95% limits of agreement for manual counting (approximately ±1.2 A-lines per image) exceeded the prespecified minimum relevant difference of one A-line. **Conclusions**: Heliox breathing was associated with a consistent, depth-dependent increase in the grayscale intensity of lung ultrasound images, suggesting that intrapulmonary gas composition contributes to image brightness. The concomitant change in A-line counts was directionally consistent but smaller than the resolution of manual counting at the imaging depth used and therefore remains inconclusive.

## 1. Introduction

Chest ultrasonography is a widely accessible imaging modality increasingly used in the assessment of pulmonary disorders. Beyond its established role in detecting pleural effusions, it is now routinely applied in the diagnosis of pneumonia and interstitial lung disease and in guiding invasive procedures, where it improves procedural safety [1,2,3]. In normally aerated lungs, ultrasound waves reflect from the pleural surface, producing horizontal artifacts known as A-lines, which indicate the presence of air beyond the pleura [4]. Reduced aeration generates vertical artifacts (B-lines), which may coalesce into a “white lung” pattern, while complete loss of aeration results in subpleural consolidations [5]. In conditions characterized by excess air, such as pneumothorax, pleural sliding is absent and the image is dominated by A-lines and multiple horizontal reverberations [6].

The diagnostic value of lung ultrasound (LUS) in the aerated lung rests on the physics of the pleural interface. Because the acoustic impedance of soft tissue exceeds that of air by more than three orders of magnitude, nearly all incident ultrasound energy is reflected at the visceral pleura, and the image beneath the pleural line consists predominantly of artifacts rather than anatomical structures [7,8,9]. A-lines arise from repeated reverberation of the ultrasound pulse between the pleural interface and the transducer and therefore appear at multiples of the skin-to-pleura distance [4,10]. Vertical artifacts (B-lines), in turn, arise in acoustic traps formed when the air-to-tissue ratio of the peripheral lung decreases (through interstitial thickening or partial filling of the alveoli with fluid), which explains their association with interstitial and alveolar disease [11,12,13,14]. Over the past decade, LUS has evolved from a qualitative, sign-based bedside approach toward quantitative analysis, including grayscale and texture analysis, spectral methods, and automated or deep-learning-based artifact detection [15,16].

Despite this progress, current understanding of artifact generation derives largely from phantom experiments, numerical simulations, and clinical observations in which the tissue and gas components of the lung change simultaneously [8,13,17]. In disease, loss of aeration, interstitial thickening, and alveolar destruction alter both the amount and the spatial arrangement of air, making it impossible to isolate the contribution of the gas itself. The influence of the physical properties of intrapulmonary gas (density, speed of sound and, consequently, acoustic impedance) on the appearance of horizontal artifacts has not been systematically investigated in humans.

Helium–oxygen mixtures (heliox) offer a safe and well-characterized means of modifying these properties in vivo. A mixture of 80% helium and 20% oxygen has approximately one-third of the density of air and a substantially higher speed of sound, and its low density has long been exploited clinically to reduce the work of breathing in airway obstruction [18]. Helium is also an established gas-phase contrast agent in pulmonary imaging, most notably in hyperpolarized helium magnetic resonance imaging of ventilation [19]. Breathing heliox therefore replaces a substantial fraction of the resident alveolar gas with a low-density gas while leaving lung structure intact, providing a controlled within-subject model for studying how gas composition affects ultrasound artifact formation.

To our knowledge, no previous human study has examined the effect of intrapulmonary gas composition on LUS artifacts. We hypothesized that replacing alveolar air with a lower-density, lower-impedance gas mixture would modify reflection at the pleural interface and, consequently, the number and brightness of horizontal artifacts. The aim of this prospective exploratory study was to evaluate the effect of breathing heliox on LUS images in healthy volunteers, using three prespecified outcome measures: the number of visible A-lines, grayscale intensity, and automatically detected horizontal reverberations. Because only the gas component of the lung is modified, this approach may provide a physical reference for the interpretation of horizontal artifacts and generate hypotheses for future studies in conditions characterized by an increased air-to-tissue ratio, such as emphysema, in which LUS interpretation remains challenging.

## 2. Materials and Methods

### 2.1. Study Design

This prospective, exploratory study used a paired within-subject design in which each participant was examined under two consecutive breathing conditions (ambient air followed by heliox) and served as their own control.

### 2.2. Study Participants

Healthy adult volunteers without respiratory symptoms were recruited. Participants were enrolled irrespective of age and self-reported sex to capture the anthropometric heterogeneity of a general adult population. Age, sex, height, and body weight were recorded, and body mass index (BMI) was calculated as body weight (kg) divided by height squared (m^2^).

#### 2.2.1. Sex and Gender Considerations

Sex was recorded as self-reported binary sex (female/male) and used as a proxy for biological sex. No participant reported discrepancies between declared and biological sex. Gender identity was not assessed, as it was not relevant to the physiological mechanisms under investigation. Sex-stratified analyses were performed in accordance with SGBA recommendations.

#### 2.2.2. Inclusion Criteria

- Healthy adults (≥18 years) without respiratory symptoms.
- Ability to provide written informed consent.
- No history of chronic pulmonary disease or acute respiratory illness at the time of enrollment.

#### 2.2.3. Exclusion Criteria

- Abnormal lung ultrasound findings at baseline.
- Lack of consent.
- Withdrawal before completion of the protocol.

The study was conducted in accordance with the Declaration of Helsinki and approved by the Bioethics Committee of the Medical University of Gdańsk (approval no. KB/381/2025). Written informed consent was obtained from all participants.

### 2.3. Study Procedure

Each participant underwent two thoracic ultrasound examinations:

1. during spontaneous breathing of ambient air,
2. during breathing of a heliox mixture (80% helium, 20% oxygen) delivered via a disposable mouthpiece with a one-way valve.

All examinations were performed with participants in the supine position. Heliox was delivered through an open-circuit demand system connected to a high-pressure cylinder (200 bar) containing a medical helium–oxygen mixture (80% He/20% O_2_). Gas pressure was reduced by a first-stage regulator to approximately 9.5 bar and delivered through a standard low-pressure hose (EN 250 compliant) to a second-stage demand regulator. With the nose occluded, participants inhaled through a hypoallergenic silicone mouthpiece fitted with a one-way exhalation valve, which vented exhaled gas to the atmosphere and prevented CO_2_ rebreathing. After baseline imaging during spontaneous breathing of ambient air, participants began breathing heliox; after ten calm tidal breaths, imaging was repeated at the same four predefined locations while heliox breathing continued throughout image acquisition. The order of conditions was fixed (air followed by heliox) and not randomized; no sham air-breathing circuit was used, and no return-to-air (washout) acquisition was performed. Ten breaths were chosen to achieve an estimated alveolar helium fraction above 60% according to the mixing model described below; the alveolar helium fraction was not measured directly.

Because direct measurement was not performed, the alveolar helium fraction was estimated using a single-compartment, homogeneous (well-mixed) model with an assumed tidal volume (VT) of 0.5 L and functional residual capacity (FRC) of 2.5 L. With each breath, a fraction VT/(FRC + VT) of the alveolar gas is replaced by inspired gas, which yields an estimated alveolar helium fraction of approximately 67% after ten breaths, according to:

(1) $$F_{n+1}=F_{i}\times \frac{\mathrm{VT}}{\mathrm{FRC}+\mathrm{VT}}+F_{n}\times \frac{\mathrm{FRC}}{\mathrm{FRC}+\mathrm{VT}}$$

The simplified form was

**F_n_** = **F_i_** × (**1** − **r^n^**) (2)

where F_n_ is the modeled alveolar helium fraction after *n* breaths, F_i_ is the inspired helium fraction (0.8), VT the tidal volume, FRC the functional residual capacity, and

**r** = **FRC**/(**FRC** + **VT**) (3)

Calculated alveolar helium fractions for breaths 1 through 19, based on this model, are presented in Table 1.

This model assumes instantaneous and complete mixing of inspired gas within a single, homogeneous alveolar compartment. In reality, inter-individual differences in breathing pattern, tidal volume, and FRC, together with regional ventilation heterogeneity and possible minor leaks around the mouthpiece, may cause the actual alveolar helium fraction to deviate from, and vary around, the modeled value. The model also neglects anatomical dead space: assuming an effective alveolar volume of 0.35 L per breath instead of 0.5 L lowers the estimate after ten breaths to approximately 58%, whereas assuming, in addition, the lower FRC expected in the supine position (2.0 L) raises it to approximately 64%. The estimate should therefore be regarded as an approximate reference point rather than a measured concentration.

### 2.4. Ultrasonography

**Figure 1:**
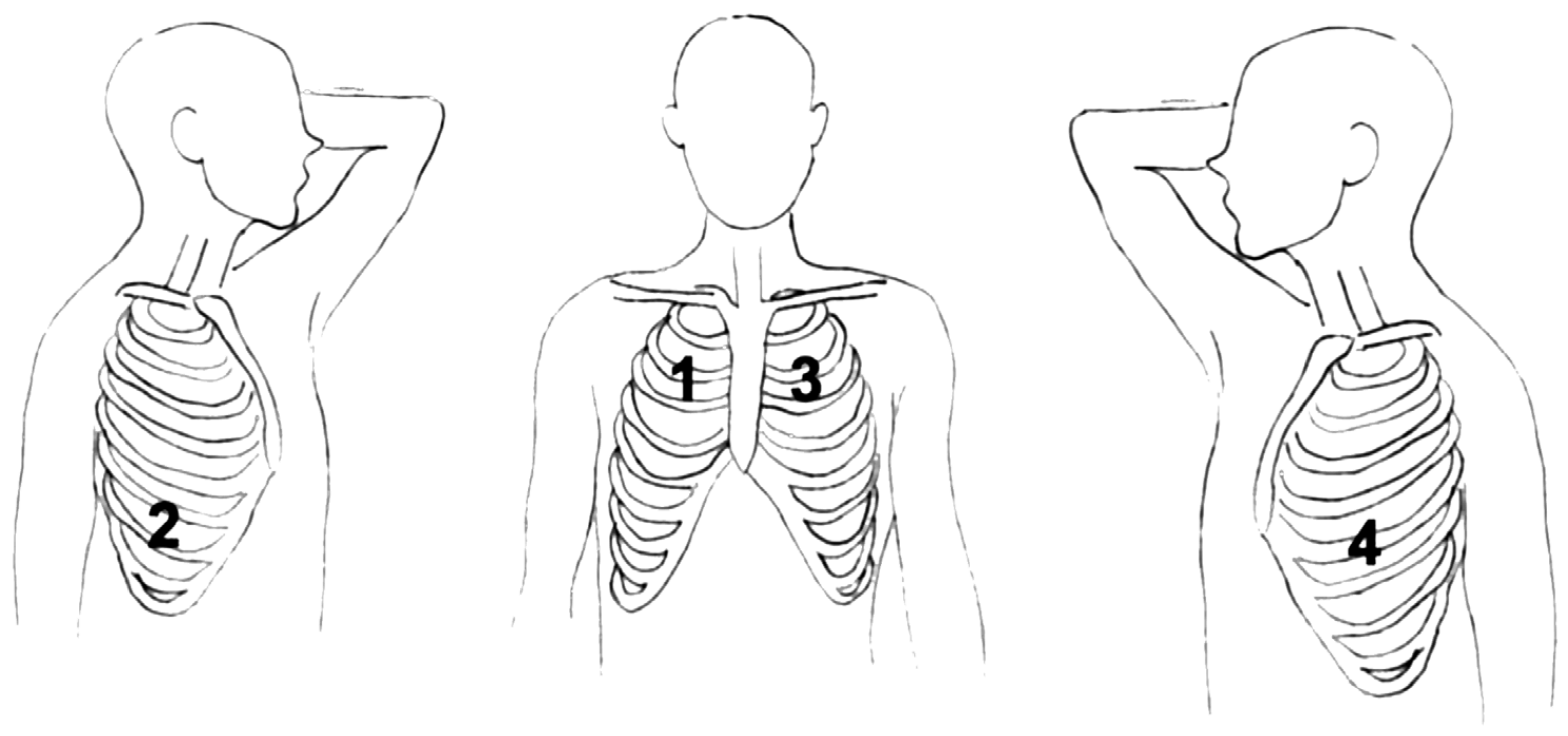
Visualization of the ultrasound probe placement sites on the volunteer’s body from the front and the side. 1, right upper anterior field; 2, right lower lateral field; 3, left upper anterior field; 4, left lower lateral field. Fields 1 and 3 were analyzed as upper and fields 2 and 4 as lower lung fields.

Examinations were performed in the supine position using standard intercostal acoustic windows in the sagittal plane (four anatomical locations) as shown in Figure 1.

Acquisition settings were identical in both breathing conditions and are listed in Table 2. Tissue harmonic imaging (THI) was enabled, whereas the image-enhancement algorithms iClear and iBeam were disabled.

**Figure 2:**
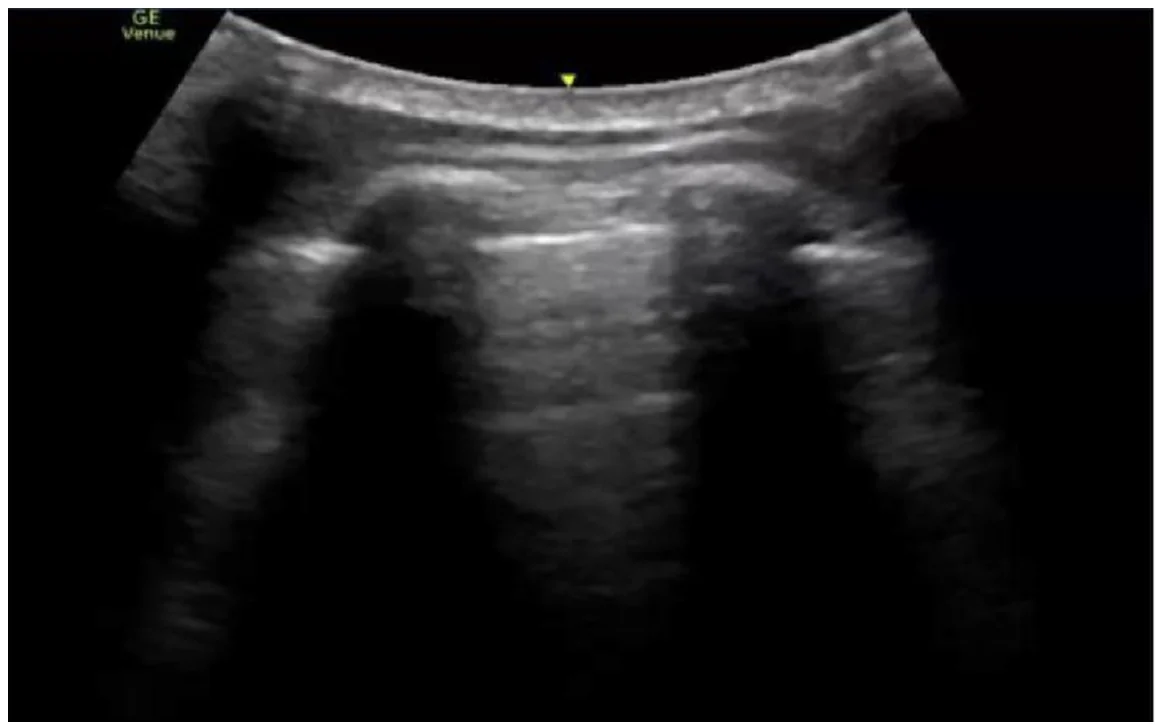
Lung ultrasound (LUS) image demonstrating the “bat sign,” characterized by two ribs and the pleural line between them.

Correct probe placement was confirmed by visualization of the “bat sign” (two ribs and the pleural line between them), as illustrated in Figure 2.

**Figure 3:**
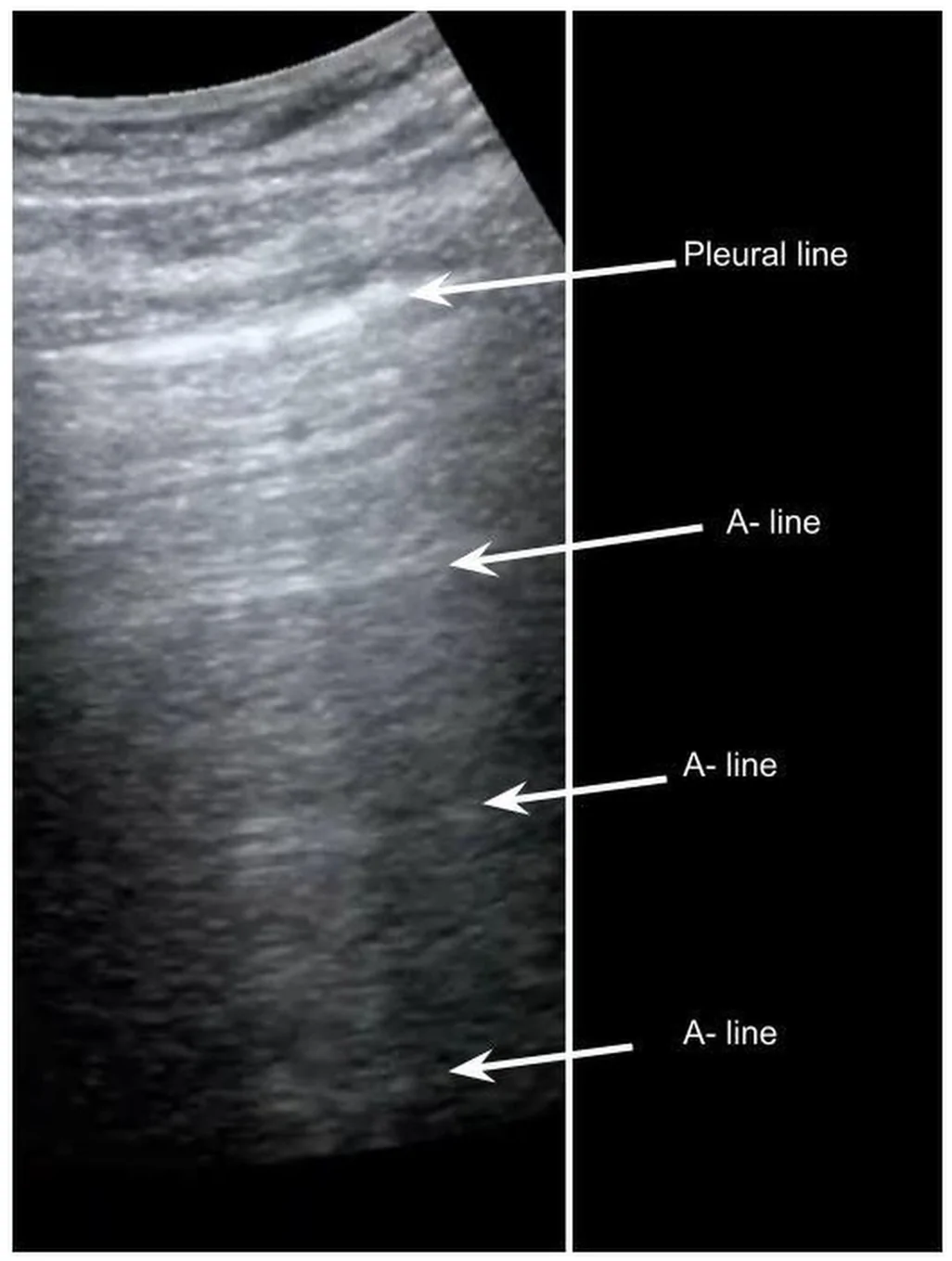
Lung ultrasound (LUS) image with marked A-lines.

After confirming normal lung aeration and the presence of A-lines, cine loops were stored for offline analysis, as shown in Figure 3.

### 2.5. A-Lines

A-lines are horizontal, hyperechoic reverberation artifacts that appear below the pleural line at regular intervals, corresponding to multiples of the transducer–pleural line distance, and, in the presence of lung sliding, indicate normally aerated lung [10]. Because no validated automated algorithm for A-line identification is available, A-lines were counted manually, whereas horizontal reverberations were quantified automatically by peak detection, as described below.

### 2.6. Image Analysis

Ultrasound images were analyzed using ImageJ (version 1.54p; National Institutes of Health, Bethesda, MD, USA). For each image, a rectangular region of interest (ROI) was drawn manually from the pleural line to the lower boundary of the field of view, encompassing horizontal reverberations. Because the ROI was narrow, the curvature of the horizontal lines resulting from the sector geometry of the convex probe was negligible within it.

All image selection and analysis were performed by a single investigator (the first author). For each acquisition, the frame with the clearest visualization of A-lines (i.e., the frame containing the greatest number of visible A-lines) was selected as the most representative segment of the recording, with preference for the central part of the image, where lateral resolution is highest. Frame selection and image analysis were not blinded to the breathing condition, but identical predefined criteria were applied to all images. Respiratory phase was not recorded and probe position was not fixed mechanically; however, all acquisitions were performed by the same operator at the same four predefined thoracic locations in accordance with standard LUS practice. The reliability of A-line counting was assessed subsequently in a blinded reassessment (Section 2.8).

#### 2.6.1. Plot-Profile Analysis

Pixel-intensity values were extracted using the Plot Profile function:

X-axis: distance from the pleural line,

Y-axis: grayscale intensity (8-bit; 0–255).

Local brightness maxima corresponding to A-lines and horizontal reverberations were detected using the Find Peaks function with the following thresholds:

minimum peak amplitude: 3, 5, 10, 20, 30, 40 grayscale units (GU; 8-bit scale),

minimum peak width: 0 px.

Detected maxima were visualized as markers and counted.

**Figure 4:**
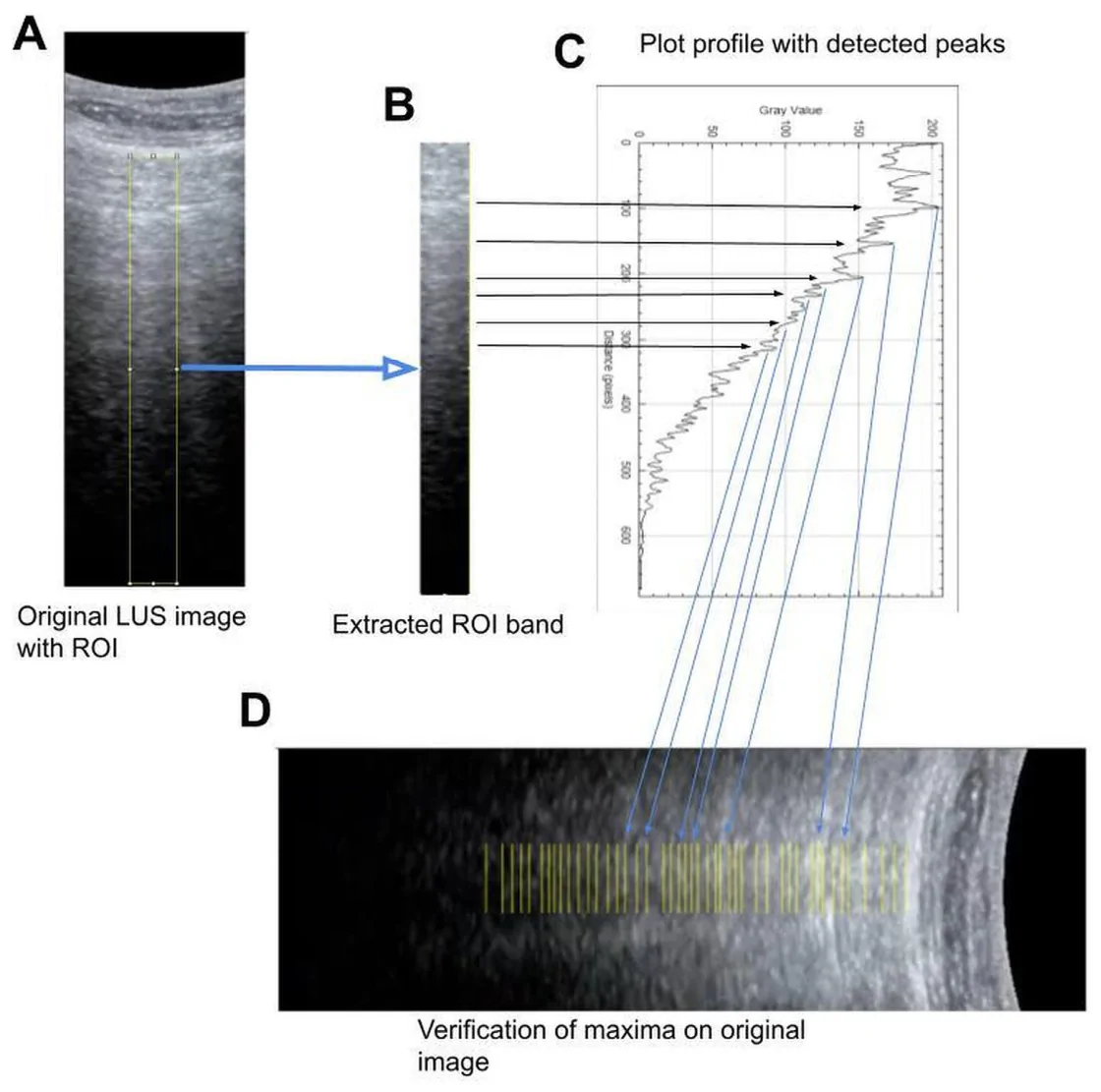
Schematic representation of the ultrasound image-processing workflow. (**A**) Original lung ultrasound image with the region of interest (ROI) extending from the pleural line to the lower boundary of the field of view. (**B**) Extracted narrow horizontal-line band used for intensity analysis. (**C**) Plot-profile generated in ImageJ, showing grayscale intensity as a function of distance and detected local maxima. (**D**) Verification of detected maxima visualized as yellow markers on the original image.

The image analysis workflow, comprising ROI selection, extraction of the horizontal-line band, generation of the plot profile with detected local maxima, and verification of peak positions on the original ultrasound image, is illustrated in Figure 4.

#### 2.6.2. Grayscale Compartment Analysis

**Figure 5:**
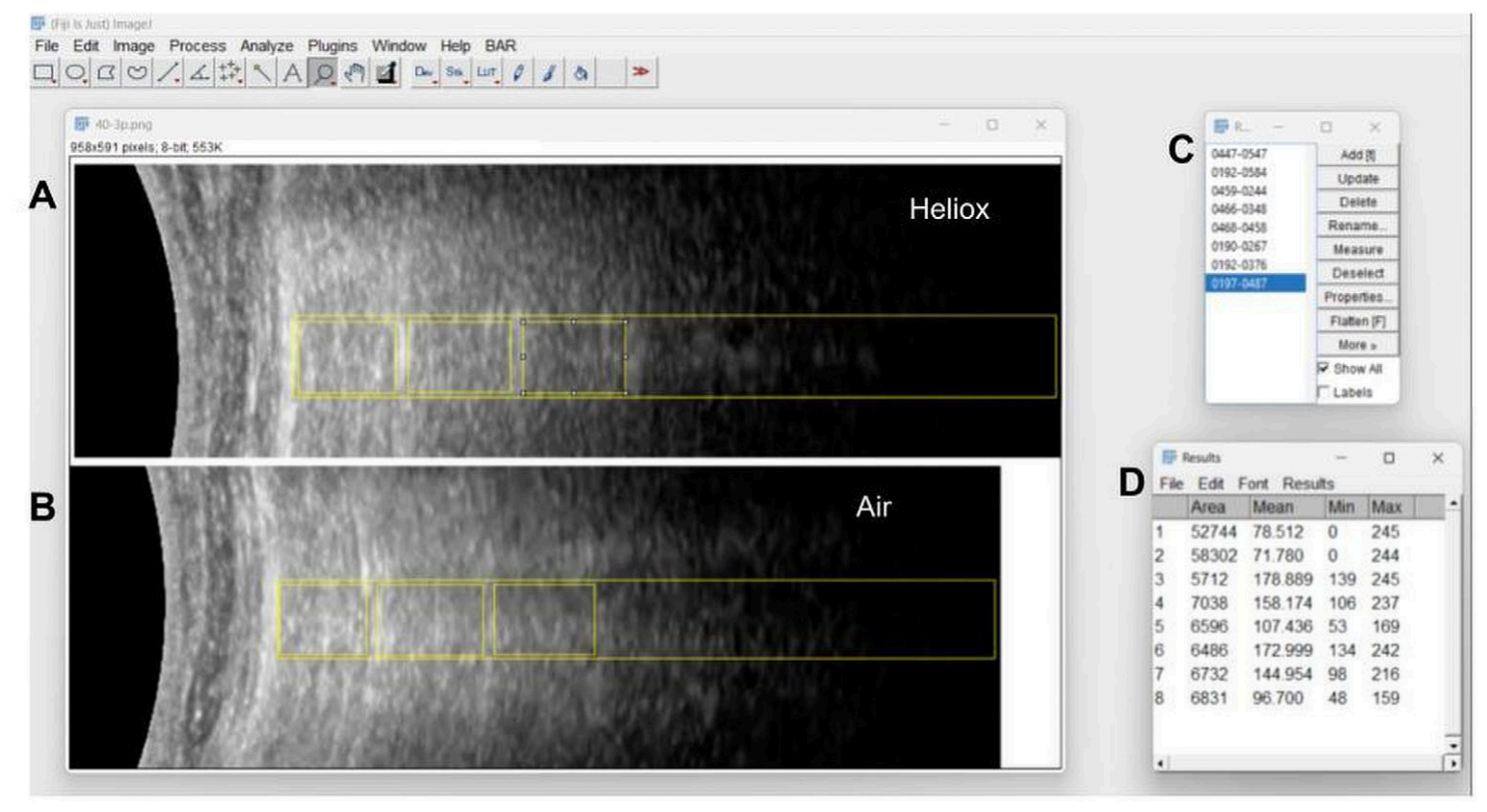
Grayscale compartment analysis in lung ultrasound images. (**A**) LUS image acquired during heliox breathing, rotated horizontally for standardized processing. The entire region from the pleural line to the lower boundary of the field of view is marked, together with individual compartments between consecutive A-lines. (**B**) LUS image acquired during ambient-air breathing, processed analogously, with delineated full-depth ROI and compartmental regions. (**C**) ROI Manager window in ImageJ showing the list of manually defined regions of interest corresponding to the full-depth ROI and individual A-line compartments. (**D**) Results window in ImageJ displaying quantitative grayscale parameters for each ROI (area, minimum, maximum, and mean grayscale intensity), with mean values used for further analysis.

Areas between consecutive A-lines were manually delineated, and mean grayscale intensity was measured within each compartment and across the entire ROI, as illustrated in Figure 5.

### 2.7. Outcome Measures

Images from the four anatomical locations acquired during ambient-air and heliox breathing were analyzed for three prespecified outcome measures:

- number of A-lines,
- number of horizontal reverberations,
- grayscale intensity (overall and compartment-specific).

**Figure 6:**
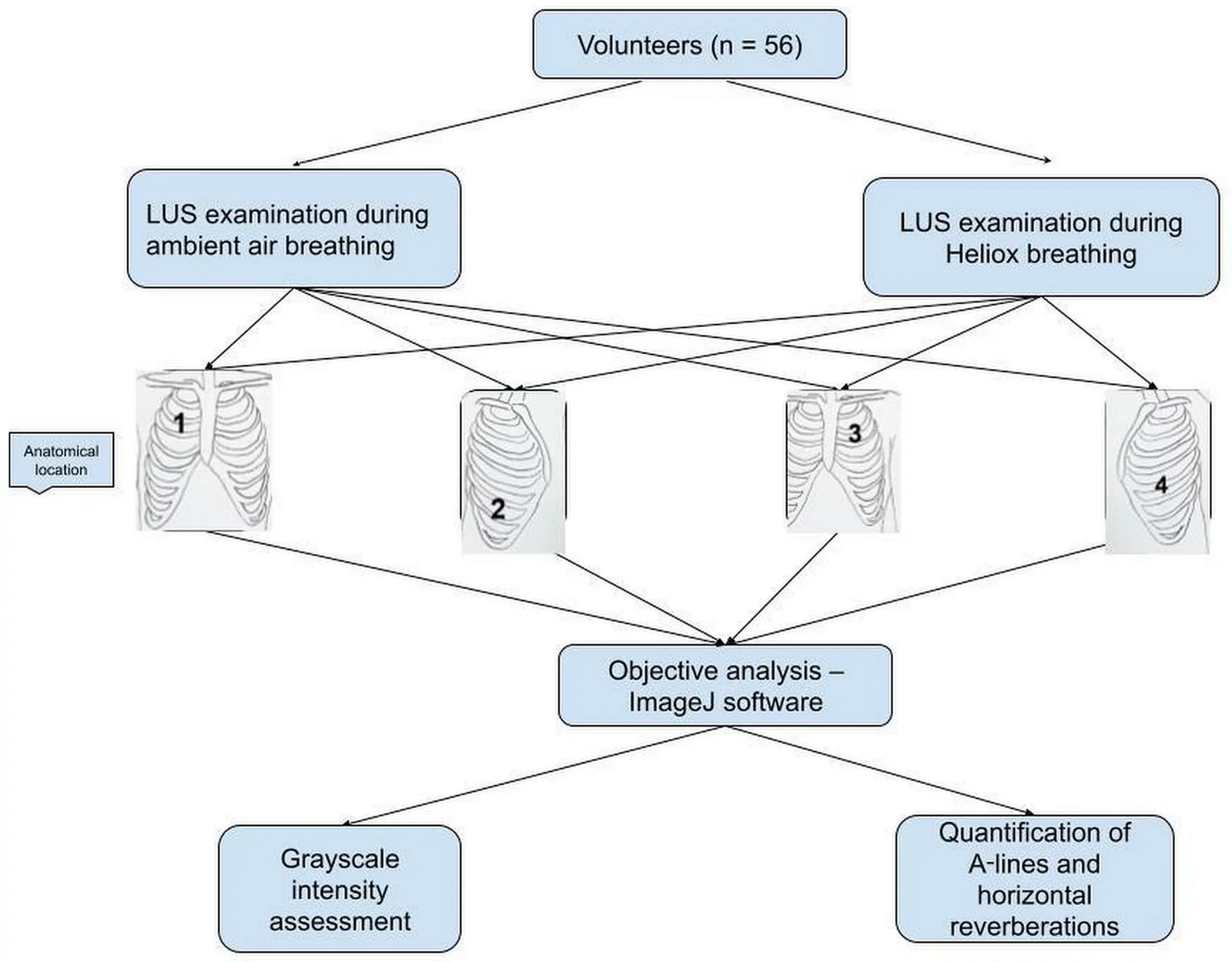
Flowchart illustrating the study protocol involving the 56 enrolled volunteers (52 included in the analysis; see Figure 7). Each participant underwent lung ultrasound (LUS) examination during ambient-air breathing and during heliox breathing. Ultrasound images were acquired from four anatomical locations (1–4), corresponding to the regions depicted in Figure 1. All images were analyzed using ImageJ software. Software-based analysis included grayscale intensity assessment and quantification of horizontal reverberations, whereas A-lines were counted manually.

A flowchart of the study protocol is shown in Figure 6.

**Figure 7:**
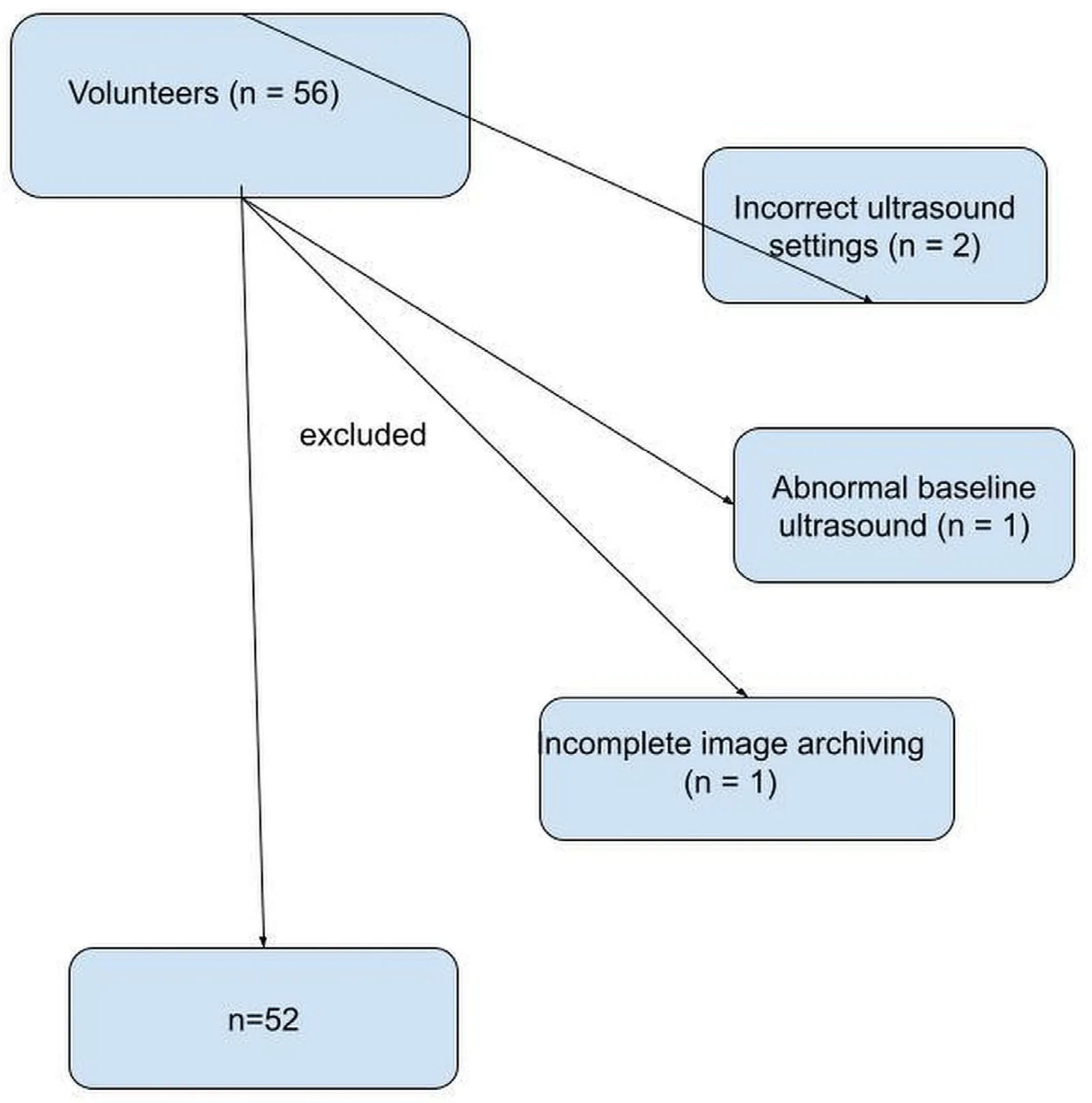
CONSORT diagram illustrating the reasons for volunteer exclusion.

#### 2.7.1. Endpoint Definitions

A-lines, defined as described above, were identified and counted manually by the first author.

Horizontal reverberations were defined as all horizontal brightness maxima detected automatically by the peak-detection procedure described above, separately at each of the six amplitude thresholds. This measure encompasses a broader category of horizontal reflections than manually identified A-lines (including low-amplitude and non-periodic reflections) and is therefore not equivalent to the A-line count.

#### 2.7.2. Compartment Definition

Grayscale compartments were delineated as rectangular subregions of the ROI extending from the pleural line toward the bottom of the image and bounded by consecutive A-line positions (compartment 1: pleural line to the first A-line; compartments 2 and 3: between consecutive deeper A-lines). When an A-line was not visible, its expected position, defined as the corresponding multiple of the transducer–pleural line distance, was used as the compartment boundary. Because the positions of A-lines are determined by the transducer–pleural line distance, which depends on chest wall thickness rather than on the inspired gas, compartment boundaries were geometrically comparable between the two breathing conditions within each participant, although their absolute size varied between participants owing to anthropometric differences. Compartment-based subdivision was preferred over fixed depth intervals because it follows the physical periodicity of the reverberation pattern.

### 2.8. Blinded Reassessment

To evaluate the reliability of manual A-line counting as the primary outcome, a representative subsample of 13 of the 52 participants (25%) was selected by stratified random sampling according to sex and BMI category (≤25 vs. >25 kg/m^2^), with proportional allocation (random seed 2026), yielding 104 images (four lung fields under each breathing condition). On a local workstation, the frames used in the primary analysis were cropped to the ultrasound sector, stripped of on-screen annotations and of other condition-identifying features, standardized in size, assigned random codes, and presented in random order, with the two images of each pair separated by at least ten positions.

The original investigator re-counted the A-lines in all coded images approximately one year after the primary analysis, applying the same predefined criteria and without access to the original counts or the allocation key (test–retest). The second author (N.B.), an investigator with extensive experience in LUS who had not participated in the primary analysis, independently counted the A-lines in the same coded images under identical conditions (inter-observer). All blinded assessments were performed on anonymized images identified only by study code; no AI tool was used at any stage of image preparation or counting.

### 2.9. Sample Size

The sample size was determined a priori for the number of visible A-lines. Assuming a paired design, a minimum relevant difference of one A-line with a standard deviation of paired differences of two A-lines (standardized effect size d = 0.5), a two-sided α of 0.05, and a power of 80%, a minimum of 34 participants was required. The final analyzed sample of 52 participants exceeded this requirement.

### 2.10. Statistical Analysis

Continuous variables are presented as mean ± standard deviation (SD) and median (range), and categorical variables as numbers and percentages. Data distribution was assessed using histograms, skewness and kurtosis, and the Kolmogorov–Smirnov and Jarque–Bera tests. Because most variables deviated from normality, non-parametric methods were used throughout.

Each participant contributed up to four lung fields under each breathing condition, and all available fields were analyzed without averaging. Because fields obtained from the same individual are not independent, paired differences (heliox minus air) across all lung fields were compared using the clustered Wilcoxon signed-rank test described by Rosner, Glynn, and Lee, with the participant as the cluster; this test uses every field-level pair while deriving its variance from between-participant variability, thereby accounting for within-participant correlation [20]. Effect estimates are reported as the mean field-level paired difference with 95% confidence intervals (CI) obtained by cluster (participant-level) bootstrap resampling with 5000 replicates, and effect sizes as r = |Z|/√*n*, where *n* is the number of non-zero paired differences (r ≈ 0.1, 0.3, and 0.5 indicating small, medium, and large effects, respectively). In addition, each lung field was analyzed separately with the Wilcoxon signed-rank test, paired within participants (n = 51–52 per field). As a sensitivity analysis, values were averaged across fields within each participant and compared with the Wilcoxon signed-rank test, with Hodges–Lehmann (HL) estimates and distribution-free 95% CI.

Three outcome measures were prespecified as co-primary: manually counted A-lines, grayscale intensity, and automatically detected horizontal reverberations; the A-line count was used for the sample-size calculation. A Bonferroni correction was applied across these three outcomes (adjusted α = 0.0167). Within outcomes comprising several measurements, Bonferroni-adjusted *p*-values were additionally calculated across the four grayscale measures (global intensity and compartments 1–3; adjusted α = 0.0125) and across the six reverberation detection thresholds (3, 5, 10, 20, 30, and 40 GU; adjusted α = 0.0083). Analyses by sex, BMI category (≤ 25 vs. > 25 kg/m^2^), and lung region (upper fields 1 and 3 vs. lower fields 2 and 4) were considered exploratory and were not adjusted for multiplicity; for field-by-field analyses, Bonferroni-adjusted *p*-values across the four lung fields are reported in addition to unadjusted values.

Because statistical significance within one subgroup but not another does not demonstrate a difference between subgroups, effect modification was tested formally with permutation tests (10,000 permutations) that preserved the clustering of fields within participants: for sex and BMI category, participant labels were permuted between subgroups, and for lung region, the lower-versus-upper contrast was sign-flipped within participants. The association between BMI and the mean change in A-line count per participant was assessed with Spearman’s rank correlation. Reliability of manual A-line counting was quantified with the intraclass correlation coefficient (ICC; ICC(3,1), two-way mixed, consistency, single measurement, for test–retest and ICC(2,1), two-way random, absolute agreement, single measurement, for inter-observer agreement), the quadratic-weighted kappa coefficient (κw), and Bland–Altman analysis (bias and 95% limits of agreement). The heliox–air difference in the blinded test–retest reading was compared with the original unblinded difference using the clustered Wilcoxon signed-rank test described above. Missing or non-measurable values (e.g., a compartment that could not be delineated) were handled by pairwise exclusion, and the number of observations is reported for each analysis. All tests were two-sided, and *p* < 0.05 was considered statistically significant; exact *p*-values are reported, except for values below 0.001. Descriptive statistics, normality tests, field-by-field Wilcoxon signed-rank and sign tests, and Spearman correlation were performed in Statistica version 13 (TIBCO Software Inc., Palo Alto, CA, USA). The clustered Wilcoxon signed-rank test, cluster-bootstrap confidence intervals, and permutation tests were implemented in Python 3.13 using the SciPy (version 1.18) and NumPy (version 2.5) libraries; the analysis code is available from the corresponding author upon request. The generative AI tool Claude (Opus 5.5; Anthropic, San Francisco, CA, USA) was used to assist in writing and verifying this code and in cross-checking the numerical values of the statistical computations against the Statistica output. The analyses were planned, performed, and interpreted by the authors, who take full responsibility for the results.

## 3. Results

Of the 56 volunteers enrolled, four were excluded: two because of incorrect ultrasound settings, one because of abnormal baseline LUS findings, and one because of incomplete image archiving (Figure 7). The final analysis included 52 participants (27 women and 25 men), who contributed 208 lung fields during ambient-air breathing; heliox images were unavailable for one field, leaving 207 paired fields. The baseline characteristics of the study population are presented in Table 3.

### 3.1. Primary Outcomes

#### 3.1.1. A-Lines

**Figure 8:**
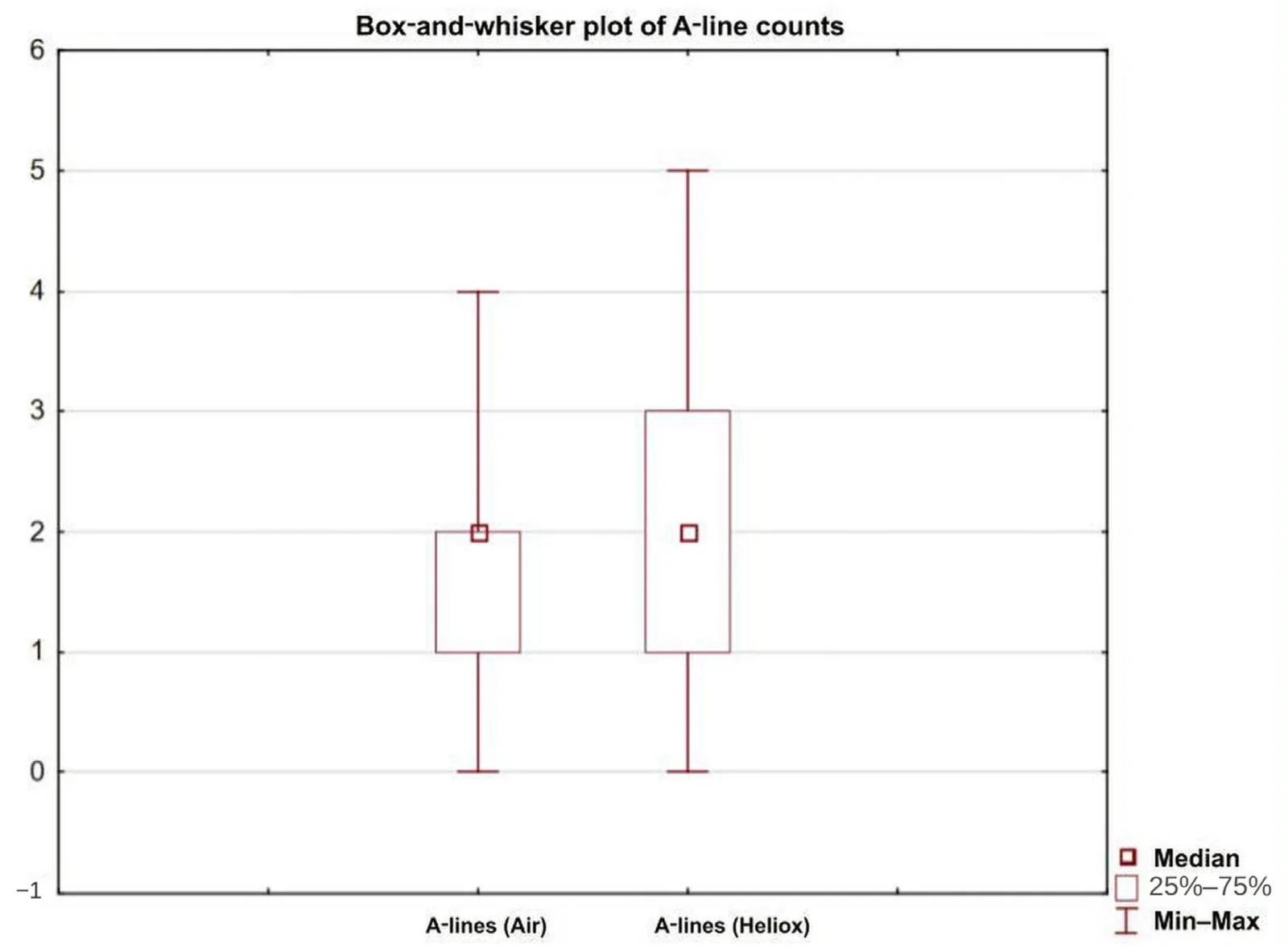
Box-and-whisker plot showing the number of A-lines during ambient-air and heliox breathing. The plot displays median values, interquartile ranges (25–75%), and minimum–maximum limits for each condition. Statistical comparisons are reported in Table 4.

Heliox breathing increased the number of visible A-lines (Table 4, Figure 8). Across all 207 paired lung fields, A-line counts increased in 86 fields, decreased in 21, and were unchanged in 100, corresponding to a mean difference of 0.45 A-lines (cluster-bootstrap 95% CI 0.30–0.62; clustered Wilcoxon Z = 4.38; *p* < 0.001; r = 0.42), which remained significant after Bonferroni correction (adjusted *p* < 0.001). In field-by-field analyses (n = 51–52 per field), the increase was significant after Bonferroni correction in fields 2 and 4 (adjusted *p* < 0.001 and *p* = 0.013, respectively) and directionally consistent in fields 1 and 3 (unadjusted *p* = 0.013 and *p* = 0.025; adjusted *p* = 0.053 and *p* = 0.100). The sensitivity analysis based on participant-averaged values yielded a concordant Hodges–Lehmann difference of 0.38 (95% CI 0.25–0.63; *p* < 0.001).

The distribution of A-line counts is shown in Figure 8.

#### 3.1.2. Reliability of A-Line Counting

Test–retest reliability of the manual A-line count was good (ICC(3,1) = 0.76, 95% CI 0.67–0.83; κw = 0.75), as was inter-observer reliability (ICC(2,1) = 0.74, 95% CI 0.63–0.81; κw = 0.73). Counts were identical in 77% of images and within one A-line in 95% in both comparisons (Table A1). The 95% limits of agreement were approximately ±1.2 A-lines per image for both the test–retest (−0.99 to +1.26) and the inter-observer analysis (−1.23 to +1.18; Figure A3), exceeding the one-A-line minimum relevant difference specified a priori for the sample-size calculation.

In the blinded test–retest, the mean heliox–air difference was 0.21 A-lines per field (95% CI 0.02 to 0.38; *p* = 0.037; Bonferroni-adjusted *p* = 0.11), compared with 0.44 (95% CI 0.23 to 0.65; *p* = 0.007; adjusted *p* = 0.021) in the original unblinded counts of the same 104 images; the difference between the two estimates was −0.23 (95% CI −0.46 to 0.00; *p* = 0.09). On average, the blinded re-count exceeded the original count by 0.25 A-lines in air images and by 0.02 A-lines in heliox images. In the independent inter-observer re-count, the mean heliox–air difference was 0.04 A-lines per field (95% CI −0.17 to 0.25). The direction of the effect was the same in all three readings, but its magnitude decreased progressively once the breathing condition was concealed.

#### 3.1.3. Horizontal Reverberations

**Figure A1:**
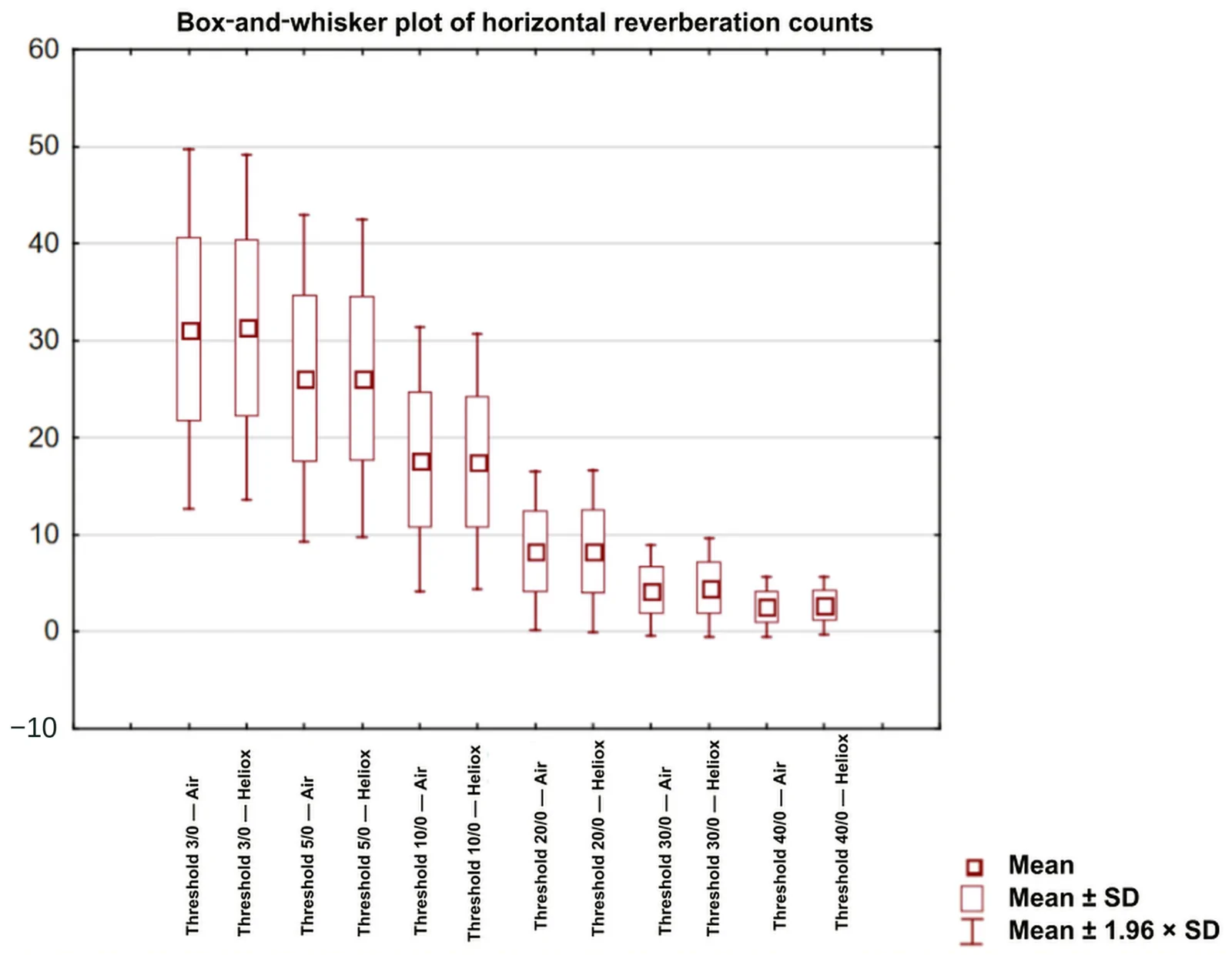
Distribution of horizontal reverberation counts at the six detection thresholds during ambient-air and heliox breathing; category labels give the minimum peak amplitude (GU)/minimum peak width (px). Squares, mean; boxes, mean ± SD; whiskers, mean ± 1.96 × SD. Statistical comparisons are reported in Table 4.

Automatically detected horizontal reverberations did not differ between ambient-air and heliox breathing at any of the six detection thresholds (all *p* ≥ 0.24; mean differences between −0.14 and 0.26; r ≤ 0.09) (Table 4). The distribution of reverberation counts across detection thresholds is illustrated in Figure A1.

Mean values of all outcome measures, stratified by sex, BMI category, and lung region, are summarized in Table 5.

#### 3.1.4. Grayscale Intensity

**Figure A2:**
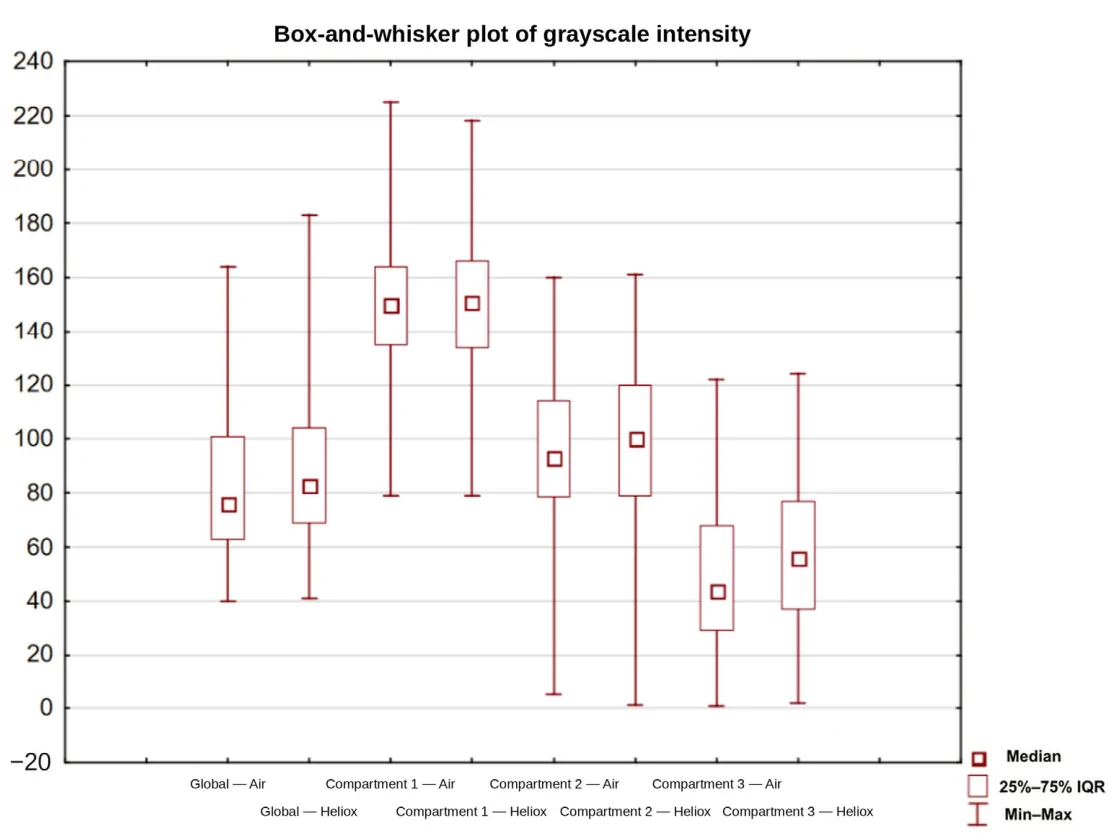
Distribution of global and compartment-specific grayscale intensity during ambient-air and heliox breathing. Squares, median; boxes, interquartile range (25th–75th percentile); whiskers, minimum–maximum. Statistical comparisons are reported in Table 4.

**Figure A3:**
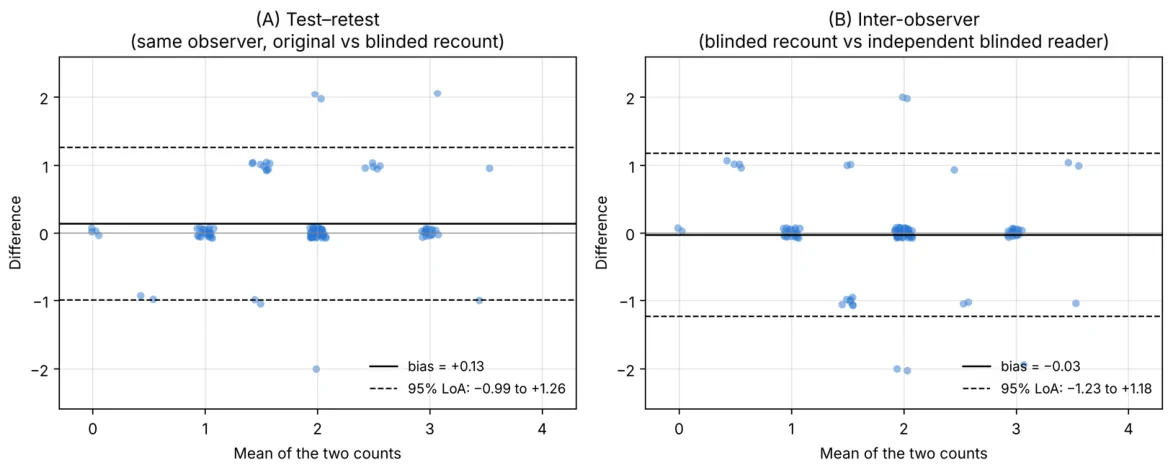
Bland–Altman plots of agreement in manual A-line counts (104 images from 13 participants). (**A**) Test–retest: blinded re-count versus original unblinded count by the same observer, performed approximately one year later; positive differences indicate a higher blinded count. (**B**) Inter-observer: independent blinded count by the second observer versus the blinded re-count; differences were calculated as the second observer’s count minus the blinded re-count, so that positive values indicate a higher count by the second observer. Symbols are jittered by up to 0.08 units in both dimensions to show overlapping observations. Solid line, mean difference (bias); dashed lines, 95% limits of agreement.

Global grayscale intensity increased during heliox breathing (mean difference 6.7 units, cluster-bootstrap 95% CI 4.1–9.2; clustered Wilcoxon Z = 4.17; *p* < 0.001; r = 0.29), with an increase in 134 and a decrease in 70 of 207 fields (Table 4). The change depended on depth: no difference was observed in compartment 1, adjacent to the pleural line (1.2 units, 95% CI −1.9 to 4.3; *p* = 0.44), whereas intensity increased in compartment 2 (6.2 units, 95% CI 2.9–9.6; *p* = 0.003; adjusted *p* = 0.012) and, most markedly, in compartment 3 (10.1 units, 95% CI 6.5–13.9; *p* < 0.001; adjusted *p* < 0.001). All grayscale effects except that in compartment 1 remained significant after Bonferroni correction. In field-by-field analyses, the increase in global intensity was significant after Bonferroni correction in field 2 (adjusted *p* < 0.001) and directionally consistent in the remaining fields (unadjusted *p* = 0.024, 0.10, and 0.13 for fields 4, 3, and 1, respectively). The sensitivity analysis based on participant-averaged values yielded a concordant HL difference of 6.4 units (95% CI 3.9–9.8; *p* < 0.001). Grayscale distributions across compartments are shown in Figure A2.

### 3.2. Sex-Stratified Analysis

In exploratory sex-stratified analyses, heliox breathing increased the number of A-lines in both women (27 participants; mean difference 0.38, 95% CI 0.17–0.60; *p* = 0.004) and men (25 participants; 0.53, 95% CI 0.30–0.77; *p* = 0.001), and increased global grayscale intensity in both sexes (women: 7.1 units, 95% CI 3.8–10.3, *p* = 0.002; men: 6.3 units, 95% CI 2.3–10.3, *p* = 0.005). Formal interaction tests did not indicate effect modification by sex (A-lines, *p* = 0.38; global grayscale, *p* = 0.77).

### 3.3. BMI-Stratified Analysis

The effect of heliox on A-line visibility differed according to BMI. In participants with a BMI ≤ 25 kg/m^2^ (34 participants, 135 fields), the number of A-lines increased (mean difference 0.64, 95% CI 0.45–0.84; *p* < 0.001), whereas in those with a BMI > 25 kg/m^2^ (18 participants, 72 fields), no significant change was observed (0.11, 95% CI −0.08 to 0.32; *p* = 0.31). The difference between BMI categories was supported by a formal interaction test (*p* = 0.001), and the mean change in A-line count per participant correlated inversely with BMI (Spearman ρ = −0.40, *p* = 0.004). In contrast, the increase in global grayscale intensity was similar in both BMI categories (BMI ≤ 25 kg/m^2^: 6.6 units, 95% CI 3.3–9.9, *p* = 0.001; BMI > 25 kg/m^2^: 6.7 units, 95% CI 2.8–11.0, *p* = 0.010; interaction *p* = 0.97). These subgroup analyses are exploratory and should be interpreted with caution.

### 3.4. Regional Analysis

In exploratory regional analyses, A-line counts increased in both the upper (fields 1 and 3; mean difference 0.38, 95% CI 0.18–0.58; *p* = 0.001) and the lower lung fields (fields 2 and 4; 0.53, 95% CI 0.35–0.72; *p* < 0.001), without significant regional effect modification (interaction *p* = 0.22). In contrast, grayscale changes in the deeper compartments were confined to the lower lung fields: in compartment 2, intensity increased by 11.2 units (95% CI 7.0–15.3; *p* < 0.001) in the lower fields but not in the upper fields (1.2 units, 95% CI −3.9 to 6.2; *p* = 0.70; interaction *p* = 0.006), and in compartment 3 by 16.4 units (95% CI 10.9–21.8; *p* < 0.001) in the lower fields versus 3.4 units (95% CI −1.5 to 8.3; *p* = 0.26) in the upper fields (interaction *p* < 0.001). Global intensity increased more in the lower (9.1 units, 95% CI 5.4–12.4; *p* < 0.001) than in the upper fields (4.2 units, 95% CI 0.5–7.9; *p* = 0.027), although this interaction did not reach statistical significance (*p* = 0.078). At the 10 GU detection threshold, horizontal reverberations in the upper fields were nominally less frequent during heliox breathing (−0.96, 95% CI −2.07 to 0.13; *p* = 0.024), but this difference did not remain significant after correction for the six detection thresholds (adjusted *p* = 0.15).

### 3.5. Visualization of Grayscale Profiles

**Figure 9:**
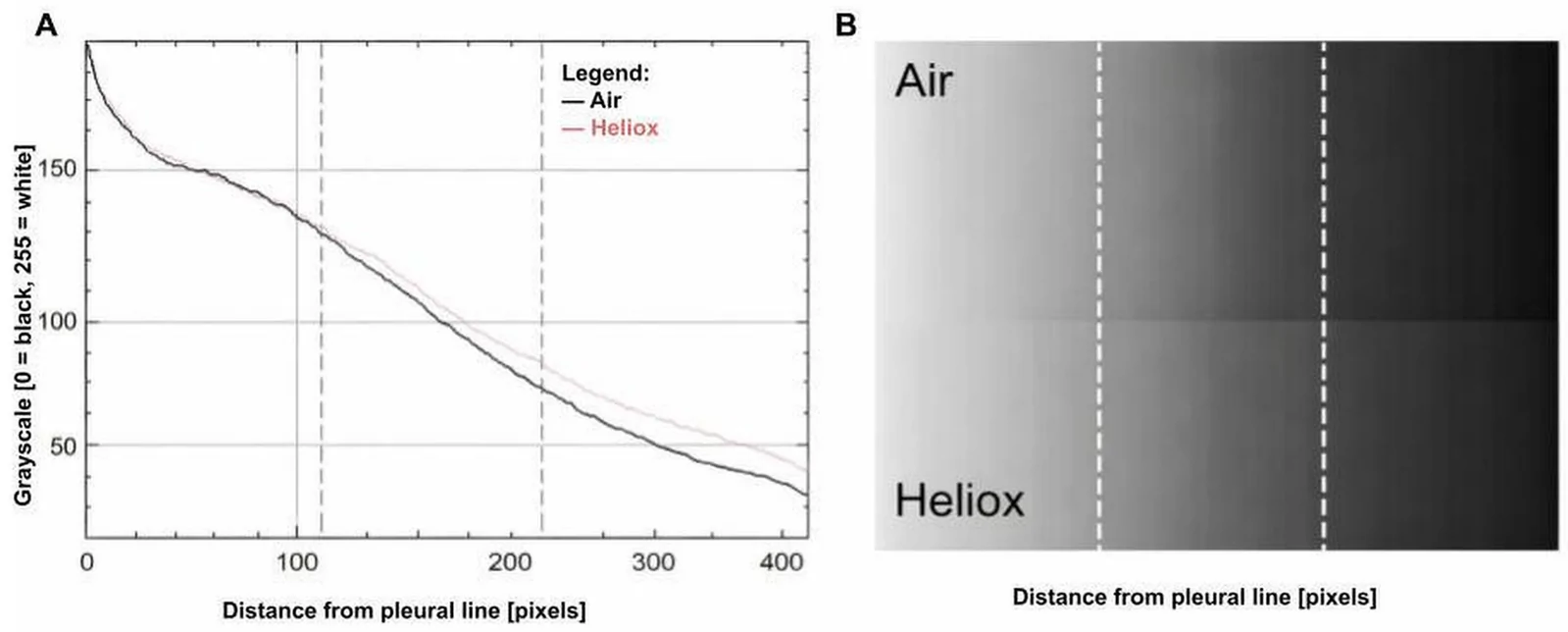
(**A**) Averaged grayscale-intensity profiles for both breathing conditions (ambient air and heliox). The black curve represents the mean grayscale distribution during ambient-air breathing, and the red curve represents the distribution during heliox breathing. Profiles are plotted across normalized depth to allow direct comparison of signal-attenuation patterns. (**B**) Grayscale visualization reconstructed from the averaged intensity profiles. The mean profiles for both conditions were converted into a horizontal grayscale map to facilitate visual comparison of grayscale distribution across the scanned region. Vertical dashed lines indicate the compartment boundaries used for quantitative analysis, as described in Section 2.7.2.

Mean intensity profiles for both breathing conditions were averaged and plotted together (Figure 9A). To illustrate grayscale differences, the averaged profiles were transformed into a grayscale map (Figure 9B).

## 4. Discussion

In this study, breathing a low-density helium–oxygen mixture was associated with measurable changes in the ultrasound appearance of the normally aerated lung: higher grayscale intensity, most pronounced in the deeper image compartments, and a smaller, direction-consistent change in the number of visible A-lines. Automatically detected horizontal reverberations did not differ between conditions. Because short-term heliox inhalation does not alter lung structure, the grayscale finding is consistent with a contribution of the acoustic properties of the alveolar gas to image brightness; the A-line finding was limited by the measurement resolution of manual counting at the imaging depth used (see below).

The physical behavior of ultrasound at tissue–gas interfaces provides a plausible framework for these observations. At boundaries between media with markedly different acoustic impedance, a large proportion of the incident energy is reflected, and the fraction that penetrates the aerated lung undergoes scattering and progressive attenuation [11]. Given the effective impedance of aerated lung parenchyma (0.18 MRayl) and of soft tissue (1.62 MRayl) [21], approximately 36% of the incident energy is transmitted across the soft tissue–lung interface, whereas at a soft tissue–air interface transmission falls to approximately 0.1%.

The acoustic impedance of helium is very low compared with that of air or biological tissues; for gaseous helium at 20 °C, the product of density and speed of sound is approximately 0.00017 MRayl [22]. Using ideal-gas approximations, we estimated the acoustic impedance of the inspired heliox mixture (80% He/20% O_2_) at approximately 0.00025 MRayl, compared with approximately 0.00041 MRayl for air. At a pure soft tissue–gas interface, both values imply reflection of approximately 99.9% of the incident energy, so that reflection at a soft tissue–heliox boundary is only marginally greater than at a soft tissue–air boundary. Heliox differs from air not only in density but also in the speed of sound (approximately 635 m/s, estimated from the ideal-gas relation c = √(γRT/M), vs. 343 m/s for air at 20 °C) and in the adiabatic index [22,23]; the observed ultrasound changes therefore cannot be attributed specifically to gas density. These calculations assume ideal-gas behavior and homogeneous mixing and do not account for the microstructure of the lung parenchyma; they are theoretical estimates intended to illustrate the order of magnitude of impedance differences rather than measured values.

The above estimate assumes a direct soft tissue–gas interface; however, the pleural line in vivo corresponds to the boundary between soft tissue and the aerated lung parenchyma, which includes a small but non-negligible volume fraction of tissue (alveolar walls, septa, vessels). Accounting for this parenchymal fraction yields an effective lung impedance several hundred times higher than that of the gas alone and, correspondingly, a substantially lower reflection coefficient at the pleural interface, as estimated below. To approximate the behavior of the aerated lung as a whole, we assumed that the tissue volume fraction of the parenchyma remains unchanged and that only the gas phase is replaced, so that the effective impedance of the lung scales with the ratio of the gas-phase impedances. Under this simplified and unvalidated assumption, the effective impedance of a lung in which the alveolar gas is entirely replaced by the inspired heliox mixture (80% He/20% O_2_; a theoretical upper bound) would be approximately 0.11 MRayl, which would increase the reflection coefficient at the soft tissue–lung interface from approximately 64% for an air-filled lung to approximately 76%. If the estimated alveolar helium fraction of approximately 67% after ten breaths is used instead of the inspired fraction, the corresponding estimates are approximately 0.00029 MRayl for the alveolar gas, 0.13 MRayl for the lung, and a reflection coefficient of approximately 73%. Higher reflectivity at the pleural interface would be expected to increase the amplitude of the reverberating pulse and thereby the number of A-lines visible above the noise floor, which is consistent with the overall direction of our observations; however, this mechanism remains a hypothesis that requires experimental validation, for example in phantoms or ex vivo lung preparations. Effective-medium models of bubbly media, in which the compressibility of the mixture is governed by the gas phase and its density by the tissue phase, would predict only a small change in effective lung impedance with heliox, driven by its higher adiabatic index rather than its lower density and opposite in sign; the estimates above are therefore illustrative and do not establish the direction or magnitude of the effect.

The increase in A-line visibility was modified by body habitus: it was pronounced in participants with a BMI ≤ 25 kg/m^2^, small and non-significant in those with higher BMI, and the difference was supported by a formal interaction test. Because this subgroup analysis was exploratory and based on the unblinded primary counts, it should be regarded as hypothesis-generating. Thicker subcutaneous fat, muscle, or breast tissue attenuates and scatters the ultrasound beam on both its incident and returning paths, reducing the amplitude of successive reverberations [7]. Moreover, because A-lines occur at multiples of the skin-to-pleura distance, a thicker chest wall increases the spacing between A-lines, so that fewer of them fall within the fixed imaging depth of 10 cm used in this study. Both mechanisms may limit the extent to which increased pleural reflectivity can translate into additional visible A-lines in individuals with higher BMI. By contrast, grayscale changes did not differ between BMI categories, suggesting that this quantitative measure may be less sensitive to chest wall thickness than the count of discrete artifacts.

The depth-dependent increase in grayscale intensity, absent immediately beneath the pleural line and most pronounced in the deepest compartment, is consistent with a greater proportion of energy being retained in the reverberation path between the pleura and the transducer. Even when more energy is reflected at the pleural interface, the reverberating pulse continues to lose energy through absorption and scattering in the chest wall [24,25]; higher initial reflectivity would therefore be expected to become apparent predominantly at greater depths, where the signal during air breathing approaches the noise floor.

In contrast, grayscale intensity in the compartment adjacent to the pleural line did not change. Near the pleura, image brightness is dominated by the strong specular reflection of the pleural interface itself, and any additional effect of heliox on small-scale scattering from subpleural structures (e.g., through the longer acoustic wavelength in the gas phase) would be expected to be small.

Three assessments of A-line counts in the same 104 images (the original unblinded count, a blinded test–retest by the same observer, and an independent blinded count by a second observer) showed the same direction of effect but a progressively smaller magnitude as knowledge of the breathing condition was removed (0.44, 0.21, and 0.04 A-lines per field, respectively). Agreement between the measurements was good (ICC 0.74–0.76), but the 95% limits of agreement for manual A-line counts (±1.2 A-lines per image) exceeded the one-A-line minimum relevant difference specified a priori. These two findings together indicate that the measurement resolution of visual A-line counting, within the range of counts observed in this cohort (0–5 per image at a 10 cm imaging depth), is insufficient to reliably resolve sub-integer differences. The A-line finding of the present study should therefore be interpreted as directionally consistent with the hypothesis but methodologically indeterminate; this question cannot be resolved with the present data (see Section 4.2).

The absence of a change in automatically detected horizontal reverberations is a separate finding and does not corroborate the A-line result. The automated algorithm was not designed to replace A-line counting; it was intended to quantify the broader population of horizontal brightness maxima (including, but not limited to, A-lines), to test whether this larger pool of reflections would differ between conditions, which it did not. The algorithm counts every local brightness maximum above a given threshold, including speckle and low-amplitude, non-periodic reflections (approximately 31 per image at 3 GU, compared with fewer than three visible A-lines), and does not use the spatial periodicity that defines A-lines. It therefore does not substitute for an A-line detection algorithm, and no inference about A-lines should be drawn from this measurement.

Changes in the deeper grayscale compartments were confined to the lower lung fields, whereas the increase in A-lines did not differ significantly between regions. One possible explanation is the regional gradient of ventilation: basal regions adjacent to the diaphragm receive a greater share of tidal ventilation [26] and may therefore achieve a higher alveolar helium fraction after a fixed number of breaths, whereas less-ventilated regions retain a larger proportion of resident gas. Regional differences in chest wall thickness and in the acoustic window may also have contributed. These explanations remain speculative, because regional gas concentrations were not measured, and should be tested in future studies using direct measurements of regional gas distribution or advanced modeling.

Analyzing all lung fields with a cluster-adjusted test avoided treating fields from the same participant as independent observations [20], and the field-by-field and participant-averaged sensitivity analyses yielded concordant results.

From a clinical perspective, the present findings do not establish diagnostic utility. Their relevance lies in suggesting that the composition of intrapulmonary gas, and not only the amount and distribution of tissue, can influence horizontal artifacts and image brightness. This supports the use of quantitative grayscale measures as physically meaningful descriptors of the aerated lung, and it raises the hypothesis that conditions with an increased air-to-tissue ratio could exhibit analogous changes. In emphysema, however, alveolar walls are destroyed and air spaces enlarged, and the lung parenchyma (composed of alveoli, septa, air, surfactant, and interstitial components) is structurally heterogeneous [12,17]; non-specular reflections from alveolar clusters, partial penetration near septa, and alterations in vascular architecture may substantially modify the ultrasound appearance. Heliox inhalation in healthy lungs alters the gas phase but does not reproduce these structural changes; extrapolation to emphysema is therefore speculative.

The effects of heliox were observed in both women and men, and formal interaction tests did not indicate effect modification by sex; because sex was self-reported and the study was not powered for sex-specific comparisons, this finding should be interpreted with caution.

### 4.1. Limitations

This study has several limitations. First, the order of conditions was fixed (ambient air followed by heliox), no sham circuit was used, and no return-to-air acquisition was performed, so time-related effects, changes in breathing pattern induced by the apparatus, and differences in probe repositioning cannot be excluded as contributors.

Second, the alveolar helium fraction was modeled rather than measured and probably varied between participants and lung regions; such variability would most likely bias the effects toward the null. Because heliox changes density, speed of sound, and the adiabatic index together, the effects cannot be attributed to a single gas property, and the impedance estimates are theoretical.

Third, LUS is inherently operator-dependent, and manually counted A-lines were obtained by a single investigator who was not blinded to the breathing condition in the primary analysis; respiratory phase and probe position were not formally controlled. Because frames were selected to show the greatest number of visible A-lines and the study hypothesis predicted an increase during heliox breathing, unblinded frame selection and counting could have inflated the apparent effect. The blinded test–retest and inter-observer reassessment reported here showed that the direction of the effect was preserved under blinded conditions, but its magnitude was substantially attenuated, and manual counting lacked the resolution to detect sub-integer differences. Blinded frame selection was not performed, and the second observer counted on frames pre-selected under unblinded conditions, so this analysis does not address bias at the stage of frame selection. Grayscale measurements, although derived from automated per-pixel analysis, depend on manually placed ROIs and cannot be regarded as fully observer-independent; moreover, the reproducibility of ROI placement was not formally assessed, although the quantization step of the 8-bit scale (1 GU) is small relative to the observed effect.

Fourth, fixed acquisition settings and a 10 cm imaging depth were used in all participants, which ensured comparability between conditions but may have reduced artifact visibility in participants with a thicker chest wall; abundant breast tissue in some women also impaired image quality. THI was enabled in both conditions; because it suppresses part of the reverberation signal, absolute A-line counts are not directly comparable with those of studies using fundamental imaging.

Fifth, the automated peak-detection method was not validated for A-line identification, grayscale compartments varied in size between participants, and subgroup and regional analyses were not adjusted for multiplicity. Finally, only healthy, predominantly young volunteers were studied, and the findings cannot be extrapolated to patients with lung disease.

### 4.2. Future Directions

A subsequent study designed specifically to resolve the A-line question would benefit from two methodological changes. First, imaging depth should be increased beyond the 10 cm used here, so that the expected A-line count per image spans a broader range than 0–5 and the minimum relevant difference substantially exceeds the measurement resolution of manual counting. Second, acquisition should be continuous rather than before-and-after, with ultrasound recording during ongoing heliox administration and during the subsequent washout phase, so that the temporal profile of the effect can be observed as alveolar gas composition changes dynamically. Repeating the protocol in fundamental imaging mode would also help to separate the contribution of tissue harmonic imaging from that of the change in gas composition. More broadly, methodological robustness would be strengthened by standardized acquisition protocols, blinded multi-reader analysis, and the development of validated automated algorithms capable of distinguishing true A-lines from non-specific horizontal reflections [15,16].

## 5. Conclusions

In healthy adults, breathing a low-density helium–oxygen mixture was associated with a consistent increase in the grayscale intensity of lung ultrasound images, most pronounced in the deeper image compartments. The number of visible A-lines changed in the same direction in unblinded and blinded readings, but the change was smaller than the resolution of manual counting at the imaging depth used and should be regarded as hypothesis-generating. Automatically detected horizontal reverberations did not differ between conditions; because the algorithm was not designed to identify A-lines, this result does not inform the A-line question. Grayscale intensity is therefore the best-supported finding of this study and is consistent with a contribution of intrapulmonary gas composition to image brightness in lung ultrasound. Whether gas composition also alters the number of visible A-lines should be addressed in a dedicated study with greater imaging depth.

## Figures and Tables

**Table 1:** Modeled helium fraction in the alveoli across consecutive breaths (VT = 0.5 L; FRC = 2.5 L; heliox 80% He).

Number of Breaths	Modeled Alveolar Helium Fraction (%)
1	13.33%
2	24.44%
3	33.70%
4	41.42%
5	47.85%
6	53.21%
7	57.67%
8	61.39%
9	64.50%
10	67.08%
11	69.23%
12	71.03%
13	72.52%
14	73.77%
15	74.81%
16	75.67%
17	76.39%
18	77.00%
19	77.50%

**Table 2:** Ultrasound system and image acquisition settings (identical in both breathing conditions).

Parameter	Value
System	Mindray TE7 (Mindray, Shenzhen, China)
Transducer	Convex 5–1 MHz
Preset	Abdomen
Tissue harmonic imaging (THI)	Enabled (identical in both breathing conditions)
Other image-enhancement (post-processing) algorithms	Disabled (iClear, iBeam)
Frequency	6.0 MHz (displayed harmonic frequency)
Dynamic range	150 dB
Overall gain	60
Frame rate	26 fps
Mechanical Index (MI)	<1.0
Thermal Index (TIS)	<0.4
Depth	10 cm
Focal point	Positioned at the pleural line

**Table 3:** Baseline characteristics of the study population.

Characteristic	Unit	Mean ± SD (*n*)	Median	Range
Age	years	30 ± 10	24.5	18–58
Sex—female	*n* (%)	27 (52%)	–	–
Sex—male	*n* (%)	25 (48%)	–	–
Height	cm	175 ± 9	175.5	155–192
Body weight	kg	74 ± 15	72	49–107
BMI	kg/m^2^	24 ± 3	24	18–33

**Table 4:** Paired comparisons of lung ultrasound outcome measures between ambient-air and heliox breathing across all lung fields (clustered Wilcoxon signed-rank test with the participant as the cluster; 52 participants, up to 207 paired fields).

Outcome Measure	Air, Mean ± SD	Heliox, Mean ± SD	Mean Difference (95% CI)	Z	*p* (p_adj_)	r
A-lines, *n*	1.66 ± 0.78	2.12 ± 1.01	0.45 (0.30 to 0.62)	4.38	<0.001 (<0.001)	0.42
Grayscale, global (GU)	81.3 ± 24.5	88.0 ± 25.3	6.7 (4.1 to 9.2)	4.17	<0.001 (<0.001)	0.29
Grayscale, compartment 1 (GU)	147.6 ± 23.4	148.8 ± 23.2	1.2 (−1.9 to 4.3)	0.77	0.44 (1.00)	0.05
Grayscale, compartment 2 (GU)	94.1 ± 28.1	100.3 ± 27.2	6.2 (2.9 to 9.6)	2.96	0.003 (0.012)	0.21
Grayscale, compartment 3 (GU)	47.9 ± 26.5	58.0 ± 27.1	10.1 (6.5 to 13.9)	4.15	<0.001 (<0.001)	0.31
Reverberations, ≥3 GU	31.1 ± 9.4	31.3 ± 9.1	0.21 (−1.02 to 1.42)	0.06	0.95 (1.00)	0.00
Reverberations, ≥5 GU	26.1 ± 8.5	26.1 ± 8.4	0.04 (−1.12 to 1.19)	−0.25	0.80 (1.00)	0.02
Reverberations, ≥10 GU	17.7 ± 6.9	17.5 ± 6.7	−0.14 (−1.12 to 0.83)	−0.97	0.33 (1.00)	0.07
Reverberations, ≥20 GU	8.3 ± 4.2	8.3 ± 4.3	0.05 (−0.60 to 0.74)	−0.15	0.88 (1.00)	0.01
Reverberations, ≥30 GU	4.3 ± 2.4	4.5 ± 2.6	0.26 (−0.16 to 0.68)	1.05	0.29 (1.00)	0.08
Reverberations, ≥40 GU	2.6 ± 1.6	2.7 ± 1.5	0.13 (−0.13 to 0.39)	1.17	0.24 (1.00)	0.09

Values are field-level means ± SD; differences are heliox minus air, with 95% confidence intervals (CI) from cluster (participant-level) bootstrap resampling. Z, standardized clustered Wilcoxon signed-rank statistic (positive values indicate higher values during heliox breathing); r, effect size (|Z|/√*n*, where *n* is the number of non-zero differences); p_adj_, Bonferroni-adjusted *p*-value (A-lines: three primary outcomes; grayscale: four measures; reverberations: six detection thresholds). Compartment 3: 51 participants and 183 paired fields. GU, grayscale units.

**Table 5:** Field-level means of all measured ultrasound parameters for the whole cohort (with standard deviations) and stratified by sex (female/male), body mass index (BMI ≤ 25 and BMI > 25), and anatomical region (upper vs. lower lung fields). The table includes A-line counts, global grayscale intensity, compartment-specific grayscale intensity values (compartments 1–3; 8-bit scale, 0–255), and the number of horizontal reverberation artifacts detected across all amplitude thresholds used in the analysis (peak ≥ 3, ≥5, ≥10, ≥20, ≥30, and ≥40 grayscale units; width ≥ 0 px). Means are based on all available fields and may therefore differ slightly from the paired estimates in Table 4.

	A-Lines (Air) [*n*]	A-Lines (Heliox) [*n*]	Grayscale Intensity [0–255, 8-bit] —Air	Grayscale Intensity [0–255, 8-bit]—Heliox	Compartment 1 Grayscale Intensity [0–255, 8-bit]—Air	Compartment 1 Grayscale Intensity [0–255, 8-bit]—Heliox	Compartment 2 Grayscale Intensity [0–255, 8-bit]—Air	Compartment 2 Grayscale Intensity [0–255, 8-bit]—Heliox	Compartment 3 Grayscale Intensity [0–255, 8-bit]—Air	Compartment 3 Grayscale Intensity [0–255, 8-bit]—Heliox	Horizontal Reverberations (peak ≥ 3 GU; width ≥ 0 px)—Air	Horizontal Reverberations (peak ≥ 3 GU; width ≥ 0 px)—Heliox	Horizontal Reverberations (peak ≥ 5 GU; width ≥ 0 px)—Air	Horizontal Reverberations (peak ≥ 5 GU; width ≥ 0 px)—Heliox	Horizontal Reverberations (peak ≥ 10 GU; width ≥ 0 px)—Air	Horizontal Reverberations (peak ≥ 10 GU; width ≥ 0 px)—Heliox	Horizontal Reverberations (peak ≥ 20 GU; width ≥ 0 px)—Air	Horizontal Reverberations (peak ≥ 20 GU; width ≥ 0 px)—Heliox	Horizontal Reverberations (peak ≥ 30 GU; width ≥ 0 px)—Air	Horizontal Reverberations (peak ≥ 30 GU; width ≥ 0 px)—Heliox	Horizontal Reverberations (peak ≥ 40 GU; width ≥ 0 px)—Air	Horizontal Reverberations (peak ≥ 40 GU; width ≥ 0 px)—Heliox
Mean	1.7	2.1	81	88	148	149	94	100	49	58	31	31	26	26	18	18	8	8	4	5	3	3
SD	0.8	1	24	25	23	23	28	27	27	27	9	9	9	8	7	7	4	4	2	3	2	2
Female	1.6	2	80	87	150	153	96	102	50	58	32	31	26	26	17	17	8	8	4	4	2	2
Male	1.7	2.2	83	89	145	144	93	99	47	58	31	32	26	26	18	18	9	8	5	5	3	3
BMI ≤ 25 kg/m^2^	1.7	2.4	73	79	148	148	98	103	48	57	31	32	26	26	18	18	9	9	5	5	3	3
BMI > 25 kg/m^2^	1.5	1.6	98	104	147	150	88	95	50	59	31	31	26	26	17	16	8	8	4	4	2	2
Upper lung fields	1.8	2.1	85	90	150	148	97	98	55	57	33	31	27	26	19	17	8	8	4	4	3	3
Lower lung fields	1.6	2.1	77	86	146	150	91	102	42	58	30	31	25	26	17	18	8	9	4	5	3	3

## Data Availability

Summary data supporting the findings of this study are included in the article. The raw ultrasound images contain sensitive health information and are not publicly available because of privacy and ethical restrictions; de-identified data are available from the corresponding author upon reasonable request.

## Abbreviations

The following abbreviations are used in this manuscript:

A-lines	Horizontal reverberation artifacts at the pleural line
BMI	Body mass index
CI	Confidence interval
FRC	Functional residual capacity
GU	Grayscale units
HL	Hodges–Lehmann estimate
Heliox	Helium–oxygen gas mixture
ICC	Intraclass correlation coefficient
LoA	Limits of agreement
LUS	Lung ultrasound
MI	Mechanical index
ROI	Region of interest
SD	Standard deviation
SGBA	Sex- and gender-based analysis
THI	Tissue harmonic imaging
TIS	Thermal index (soft tissue)
VT	Tidal volume

## Appendix A

**Table A1:** Reliability of manual A-line counting in the blinded reassessment subsample (104 images from 13 participants). ICC(3,1), two-way mixed, consistency, single measurement; ICC(2,1), two-way random, absolute agreement, single measurement; κw, quadratic-weighted kappa coefficient; LoA, limits of agreement.

Comparison	ICC (95% CI)	κw	Identical Counts	Within ±1 A-Line	Bias	95% LoA
Test–retest, ICC(3,1): same observer, blinded re-count vs. original count	0.76 (0.67–0.83)	0.75	77%	95%	+0.13	−0.99 to +1.26
Inter-observer, ICC(2,1): second observer vs. blinded re-count	0.74 (0.63–0.81)	0.73	77%	95%	−0.03	−1.23 to +1.18
